# Physiotherapy-Based Rehabilitation from the Perspective of Individuals with Multiple Sclerosis: Independence and Psychological Well-Being Beyond Physical Function—A Qualitative Study

**DOI:** 10.3390/healthcare14213788

**Published:** 2026-11-06

**Authors:** Sinem Erturan Burak, Mustafa Burak, Caner Feyzi Demir

**Affiliations:** 1 Department of Physiotherapy and Rehabilitation, Faculty of Health Sciences, Fırat University, 23119 Elazığ, Türkiye; serturan@firat.edu.tr; 2 Department of Neurology, Faculty of Medicine, Fırat University, 23119 Elazığ, Türkiye; cfdemir@firat.edu.tr

**Keywords:** multiple sclerosis, physiotherapy, experience, rehabilitation, perception, qualitative research

## Abstract

**Highlights:**

**What are the main findings?**

• Most participants perceived physiotherapy-based rehabilitation as supporting their function, independence, and psychological well-being.
• Knowledgeable, friendly physiotherapists, supportive relationships, and accessible, individualized programs were perceived as facilitating participation.

**What are the implications of the main findings?**

• Physiotherapy-based MS rehabilitation should address functional, psychological, and social needs holistically.
• Individualized access and therapeutic relationships may support sustained participation and self-management.

**Abstract:**

Background/Objectives: Multiple sclerosis (MS) is a chronic, progressive neurological disease with profound effects on physical functionality, psychosocial adaptation, and quality of life. This study aims to examine the perceptions, experiences, and factors influencing participation in physiotherapy-based rehabilitation among individuals diagnosed with MS. Methods: This qualitative study was designed using a descriptive phenomenological approach. Semi-structured, in-depth interviews were conducted with 12 purposively sampled individuals with relapsing–remitting MS (10 women; mean age, 45.6 ± 6.4 years; mean Expanded Disability Status Scale score, 4.8 ± 1.5). Data were collected until code saturation was reached and analyzed using inductive thematic analysis. Results: Two overarching themes were identified: living with MS and rehabilitation in MS. Most participants perceived physiotherapy as supporting their function, independence, and psychological well-being; three perceived no change or felt worse after sessions. A friendly, knowledgeable physiotherapist, individualized exercises, and easy transportation were described as facilitators, and physical symptoms, transportation difficulties, and lack of information as barriers. Conclusions: From the participants’ perspective, physiotherapy-based rehabilitation was experienced not merely as symptomatic treatment but as support for function, independence, and psychological well-being. Structuring services around therapeutic relationships, individualized programs, and early information may facilitate participation; these perception-based findings do not establish effectiveness.

## 1. Introduction

Multiple sclerosis (MS) is a chronic, autoimmune neurological disease characterized by demyelination and neurodegeneration of the brain and spinal cord, leading to increasing disability and symptoms over time [1]. Approximately 2.8 million people worldwide are affected by MS [2]. The disease usually begins in young adulthood, between the ages of 20 and 30 years, and over time it can cause physical disability, cognitive impairment, and reduced quality of life [1]. Its clinical course is described using the phenotypes of clinically isolated syndrome, relapsing–remitting MS (RRMS), and progressive MS, which includes primary progressive and secondary progressive forms; these phenotypes are further characterized according to disease activity, based on clinical relapses and imaging findings, and disease progression [3].

The inflammatory and neurodegenerative processes seen in MS can cause physical, emotional, and cognitive impairments [4]. In a large international survey of individuals with RRMS, more than half reported that fatigue, physical weakness, balance and coordination problems, numbness or tingling, muscle stiffness, sleep problems, and memory and concentration difficulties limited their daily activities, and many reported negative effects on their work and relationships [5]. In addition, uncertainty about the course of the disease, limitations in movement, feelings of helplessness, anxiety about the future, and problems with self-confidence and self-esteem can negatively affect the psychological well-being of individuals with MS [6]. These impairments may also negatively affect a person’s daily life activities and quality of life [4,7].

Pharmacological management of MS includes disease-modifying therapies; a 2021 review reported nine classes of these therapies for relapsing forms of MS and one approved for primary progressive MS. They reduce relapses and magnetic resonance imaging lesion activity, with reductions in the annualized relapse rate ranging from 29% to 68% compared with placebo or active comparators [1]. However, there is no cure for MS, and rehabilitation is therefore a central component of care [8]; combining pharmacological treatment with neurorehabilitation is considered important in the management of the disease [9].

The complex nature and multiple symptoms of MS necessitate a multidisciplinary approach to treatment. The fundamental goal of treatment is to enable the individual to achieve the highest possible level of independence and quality of life by improving their functionality, activity, and participation [10]. National and international guidelines recommend that all individuals with MS have regular access to physiotherapy throughout the disease course, starting from the time of diagnosis [11]. Physiotherapy and rehabilitation practices have been reported to reduce pain and to improve mobility, strength, balance, and coordination, as well as mood [12].

Although studies indicate that physiotherapy treatments are generally beneficial, the evidence remains largely limited, mainly because of limited access to rehabilitation, limited research facilities and funding, the diversity of symptoms, and the complexity of study designs [13]. Furthermore, an overview of Cochrane reviews concluded that the evidence for many rehabilitation interventions remains inconclusive owing to a lack of methodologically robust trials and called for studies that report the type and intensity of interventions [14]. A review of current rehabilitation practices also noted that outcome measures have not been standardized across studies [8]. There is also insufficient consensus between the perspectives of individuals with MS on their rehabilitation and the effects reported in intervention studies [10]. In addition, studies that comprehensively examine the general feelings, emotions, and perceptions of individuals with MS regarding their illness and physiotherapy processes are quite limited in the literature. However, for an appropriate and effective rehabilitation process, it is necessary to go beyond quantitative findings and understand individuals’ own experiences, needs, and expectations. In this regard, a qualitative research approach can fill an important gap in terms of addressing the lives of individuals with MS in a more holistic way and contributing to the development of patient-centered rehabilitation services.

Previous qualitative studies have described the experiences of individuals with MS at diagnosis and in daily life, including emotional reactions, uncertainty, identity change, and social support [6,15,16,17,18,19,20,21]. Rehabilitation-focused studies found that psychological benefits were the most frequently mentioned achievements of inpatient rehabilitation in Germany [13] and that individuals in the early stages of MS in the United States often did not consider rehabilitation necessary and lacked information about it [22]; as in other qualitative MS research, most participants had RRMS [13,22,23]. However, these studies examined the illness and rehabilitation experiences separately or focused on specific situations, such as early disease or an inpatient stay [13,22]. Because access to rehabilitation depends on health-system factors, such as service availability, transportation, and affordability [24], and perceptions of rehabilitation may depend on perceived disease severity, available information, the therapeutic relationship, and possibly cultural factors [22], evidence from other contexts is needed; to our knowledge, qualitative evidence remains limited.

Unlike these studies, the present study holistically examines participants’ general feelings about their illness, their perspectives on physiotherapy and rehabilitation, and the impact of their emotional state on their physiotherapy experiences through in-depth qualitative interviews. Therefore, this study aimed to explore the perceptions and experiences of individuals with MS regarding their illness and physiotherapy-based rehabilitation, and the factors that they perceive as facilitating or hindering their participation in rehabilitation.

## 2. Materials and Methods

### 2.1. Study Design

This study was conducted using a descriptive phenomenological qualitative research method that examines the meanings individuals attribute to their lives and the essence of their experiences [23,25]. Consistent with this orientation, the data were analyzed using inductive thematic analysis undertaken within a descriptive phenomenological framework, in which patterns of meaning are identified across participants’ accounts of their lived experiences [25,26]; this combination of a phenomenological orientation and inductive thematic analysis has also been used in previous qualitative MS research [22,23]. Thematic analysis was chosen because it is a theoretically flexible method for identifying, analyzing, and reporting patterns (themes) within data [26], which made it compatible with the descriptive phenomenological aim of describing the meanings of participants’ lived experiences [25]. In practice, this orientation guided the analysis in three ways: the transcripts were read repeatedly as a whole before coding, codes were derived inductively from participants’ own descriptions rather than from a predefined framework, and the researchers’ preunderstandings were made explicit and questioned throughout the analysis (Section 2.3 and Section 2.5). Data were collected through semi-structured individual interviews. This approach is consistent with the aim of deeply examining the emotional states, perceptions of physiotherapy and rehabilitation, and experiences of individuals with MS [7]. Questions were asked in accordance with a pre-prepared interview guide, and additional questions were asked to deepen the participants’ explanations when necessary.

### 2.2. Participants

A purposive sampling method was adopted in our study. Participants were recruited from the neurology outpatient clinic of Fırat University Hospital, where eligible individuals were invited to participate by a neurologist working in the clinic; participation was voluntary. Individuals diagnosed with MS by a neurologist, aged 18 and over, possessing the cognitive and communicative competence to understand and answer interview questions, who voluntarily participated and provided written consent were included in the study. Individuals with insufficient cognitive capacity to take part in an interview, aphasia significantly affecting verbal communication, or another neurodegenerative disorder were excluded. No formal cognitive screening test was administered; cognitive and communicative competence was judged through the interviewer’s observation during the interview. A diagnosis of RRMS was not a predefined inclusion criterion, and no MS phenotype was excluded; all individuals who completed the interviews had RRMS. Similarly, the range of disability levels, assessed with the Expanded Disability Status Scale (EDSS) [27], was not purposively targeted but arose naturally from the individuals who agreed to participate; this range allowed experiences across moderate to severe disability to be captured. Ethical approval was obtained from the Fırat University Non-Interventional Research Ethics Committee (document date: 27 December 2024; document number: 29979).

In qualitative research, sampling is terminated when data begin to repeat and no new themes emerge. It has been reported that core themes may emerge in the first six interviews, and data saturation can be achieved in approximately 12 interviews [28]. Eighteen individuals receiving treatment at Fırat University Hospital were contacted, and interviews were planned with 15 individuals who agreed to participate in the study; the remaining three declined to participate. Of the 15 individuals who agreed, three later withdrew before being interviewed because of time constraints and the long distance to the interview location, and 12 individuals were interviewed. Saturation was assessed at the level of codes by examining whether each successive interview generated new codes [29]. Code saturation was reached after the 10th interview, and no new codes emerged in the 11th and 12th interviews; therefore, the 12 interviews were considered sufficient. No repeated interviews were conducted, and transcripts were not returned to participants for comment or correction.

### 2.3. Research Team and Reflexivity

All interviews were conducted face-to-face by M.B., a male assistant professor in the Department of Physiotherapy and Rehabilitation with clinical and academic experience in neurological rehabilitation. The interviewer had no prior relationship with the participants. The researchers acknowledged that their professional backgrounds might predispose them to expect positive effects of physiotherapy and that participants might be inclined to report favorable experiences to a researcher from a physiotherapy department. To limit the influence of these assumptions, the interview guide included questions on negative factors, challenges, and possible decreases in energy and endurance; two researchers coded the data independently; and the codes and themes were discussed in research team meetings and repeatedly re-examined against the data [30].

### 2.4. Data Collection

The interview guide (Table 1) was developed by the research team based on a comprehensive literature review and the researchers’ clinical and academic expertise in neurological rehabilitation. The questions focused on identifying participants’ emotional states during their illness and their experiences with physiotherapy and rehabilitation. The guide comprised open-ended questions in six domains, including questions that invited negative, neutral, and contradictory experiences (Section 2.3). The interview questions were not shared beforehand; only a brief explanation of the study’s purpose was provided.

Face-to-face interviews were conducted between 1 January and 1 December 2025, and audio recordings were made using smartphones with the written consent of the participants. Interviews, conducted in a room with only the participant and interviewer present, lasted 30–40 min. The recordings were transcribed verbatim and securely stored in accordance with confidentiality principles.

### 2.5. Data Analysis

The study was reported in accordance with the COREQ checklist [31] to increase methodological transparency (Supplementary S1). Data were analyzed according to the six stages of thematic analysis described by Braun and Clarke: familiarization with the data, generating initial codes, searching for themes, reviewing themes, defining and naming themes, and producing the report [26]. All authors familiarized themselves with the data by repeatedly reading the transcripts. Two researchers (S.E.B. and M.B.) then independently coded the transcripts line by line and generated potential themes from meaningful patterns. Themes were reviewed, defined, and supported by participant quotations, and their consistency with the dataset was assessed [26,30].

Interviews were transcribed and coded using MAXQDA 2020.2.2 (VERBI Software, Berlin, Germany) software. The analysis was inductive: codes were derived entirely from the data, and no predefined coding framework or theory was applied. Codes and themes were compared within and across interviews [32], and researchers held regular meetings to evaluate the themes; in these meetings, the codes generated independently by the two coders were compared and discussed, and the codes and themes were re-examined for consistency with the data. After code saturation was reached (Section 2.2), the final codes and themes were generated.

In the Results Section, the following quantifiers are used to indicate how many participants expressed a code: “all” (12 participants), “most” (7–11), “half” (6), “some” (3–5), and “a few” (1–2).

## 3. Results

### 3.1. Participant Characteristics

Ten participants were women and two were men, and their ages ranged from 36 to 55 years (mean, 45.6 ± 6.4 years). All participants had RRMS. EDSS scores ranged from 3 to 7 (mean, 4.8 ± 1.5; median, 4.5), and disease duration (time since diagnosis) ranged from 2 to 10.5 years (mean, 6.1 ± 3.2 years). Eight participants were married and four were single. One participant had completed lower secondary school, seven had completed high school, three held a bachelor’s degree, and one held a master’s degree. Six participants were employed (in education, healthcare, public administration, real estate, and municipal services), four were not working, and two were retired. All participants were attending physiotherapy at the time of the interview: ten attended private special education and rehabilitation centers, which provide state-funded physiotherapy for individuals with an official disability report and usually offer transportation, and two attended hospital-based physiotherapy. The total duration of physiotherapy ranged from 1 to 7 years (median, 2.5 years), and half of the participants had been receiving physiotherapy for 3 years or longer. The clinical and rehabilitation characteristics of each participant are given in Table 2. No participants wished to withdraw from the study for any reason.

### 3.2. Overview of Themes

**Figure 1:**
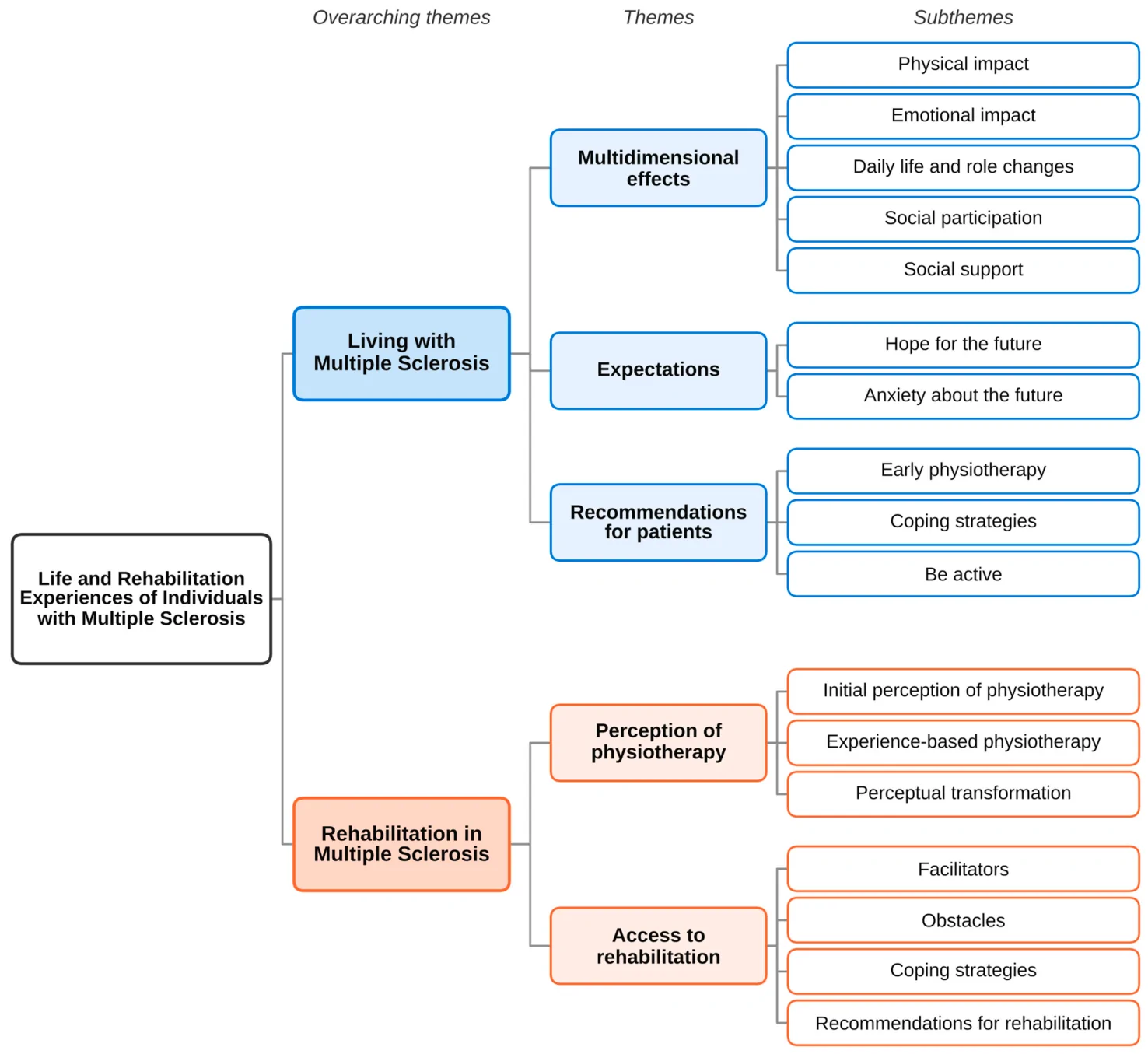
Overarching themes, themes, and subthemes identified in the study.

The data revealed two overarching themes reflecting emotional states at diagnosis and perspectives on physiotherapy and rehabilitation programs: (1) Living with MS; (2) Rehabilitation in MS. These overarching themes contained 125 and 144 coded segments (text segments assigned to codes), respectively. We presented the ‘Living with MS’ overarching theme under three themes: Multidimensional effects, Expectations, and Recommendations for patients. We presented the ‘Rehabilitation in MS’ overarching theme under two themes: Perception of Physiotherapy and Access to Rehabilitation. The subthemes, the codes with the number of participants who expressed each code, and representative quotations are shown in Table 3. The relationships between overarching themes, themes, and subthemes are shown in Figure 1. Additional quotations for each subtheme are provided in Supplementary S2.

**Table 3:** Overarching themes, themes, subthemes, codes with the number of participants (n), and representative quotations.

Theme	Subtheme	Codes (n)	Representative Quotation
**Overarching theme: Living with Multiple Sclerosis**			
Multidimensional effects	Physical impact	Fatigue (12); balance and ambulation problems (12); pain (12); sleep disorders (12); numbness/stiffness (extremity involvement) (9); worsening prognosis (6); speech difficulties (2)	“*I experience a lot of pain and spasms. I have sleep problems because of the pain*” (P01, female)
	Emotional impact	Emotional shock (12); acceptance problems (9)	“*I was very upset and cried when I first heard about it. I felt very helpless*” (P02, female)
	Daily life and role changes	Inability to perform daily tasks (12); renewal of family and work identities (10); hygiene problems (5); use of assistive devices (5)	“*Right now, I want to go and clean my house, but I can’t. I can’t clean the windows. I used to do my grocery shopping, but now I can’t*” (P05, female)
	Social participation	Social withdrawal (7); lifestyle changes (5)	“*There have been many changes since this illness. My movements aren’t like they used to be. I’ve withdrawn more into the house*” (P03, female)
	Social support	Positive social support (7); negative social support (5)	“*Thanks to this support, I don’t feel alone. Most importantly, they make me feel ‘normal’*” (P05, female)
Expectations	Hope for the future	n = 9	“*I want to get stronger, perhaps be able to move around without any physical support. Most importantly, I want to be independent*” (P07, female)
	Anxiety about the future	n = 3	“*I think I’ll get worse at this rate*” (P05, female)
Recommendations for patients	Early physiotherapy	n = 9	“*Physiotherapy is a process; even if you don’t see results immediately, you will see the difference over time. Working with a good physiotherapist is very important*” (P05, female)
	Coping strategies	Surrender to the Creator (4); acceptance and adaptation (4)	“*I hope the creator will heal it, I believe*” (P05, female)
	Be active	n = 6	“*They should continue with a suitable sport or exercise program that doesn’t overexert them*” (P06, female)
**Overarching theme: Rehabilitation in Multiple Sclerosis**			
Perception of physiotherapy	Initial perception of physiotherapy	Knowledgeable in physiotherapy (8); no knowledge of physiotherapy (4)	“*My friends and family were going for back and neck pain*” (P11, female)
	Experience-based physiotherapy	Perceived psychological, physical, and functional gains (9); no perceived change (2)	“*Thanks especially to balance exercises and stretching movements, I feel safer and the spasms have decreased*” (P06, female)
	Perceptual transformation	Understanding its effect over time/continuity (11); energy change (8)	“*Before starting physiotherapy, I had concerns about whether it would work. Now I think it’s preventing my illness from getting worse*” (P10, male)
Access to rehabilitation	Facilitators	The physiotherapist’s approach (12); improvement in well-being (12); socialization (6); ease of transportation (6); motivation (5)	“*The most important factor in my continuing treatment is my physiotherapist’s friendly and compassionate nature*” (P03, female)
	Obstacles	Physical condition (12); transportation difficulties (9); lack of information (7); psychological condition (3); the physiotherapist’s approach (2)	“*There are days when I don’t want to go to physical therapy because of fear of attacks and fatigue*” (P11, female)
	Coping strategies	Doing nothing (6); encouraging physical activity (5); Pilates, yoga, breathing exercises, etc. (2)	“*Because I have difficulty using my arm and fingers, I try to do light weight exercises, ball squeezing and releasing, and regular walking on my own*” (P11, female)
	Recommendations for rehabilitation	Individual attention and exercise expectations (11); functional exercises should be performed (11); the physiotherapist should be knowledgeable (11); well-equipped centers and appointment of physiotherapists (8); telerehabilitation centers and services (5); provision of support (3); no suggestions (1)	“*Because of my vertigo, I shouldn’t do prone exercises. A physiotherapist should create a personalized exercise program for me*” (P10, male)

n: number of participants who expressed the code (of 12); within each subtheme, codes are listed in descending order of n. Additional quotations are provided in Supplementary S2.

### 3.3. Living with MS

#### 3.3.1. Multidimensional Effects

Physical Impact: All participants in our study reported frequently experiencing fatigue, pain, sleep problems, and balance and walking problems after being diagnosed with MS. Most participants reported experiencing numbness, tingling, stiffness, or decreased muscle strength in their upper or lower extremities. These symptoms were often intertwined; for example, pain disrupted sleep, and fatigue increased the need for rest: “*I experience a lot of pain and spasms. I have sleep problems because of the pain*” (P01, female); “*I experience extreme fatigue and need more sleep*” (P09, male). A few participants reported speech difficulties, while half stated that their condition initially improved but worsened over time, describing how their limitations had accumulated as the disease progressed: “*Initially, I wasn’t physically affected much, but as the disease progressed, my hand became numb, I couldn’t do work, and I had balance problems*” (P11, female).

Emotional Impact: All participants reported experiencing emotional turmoil upon receiving their MS diagnosis, including crying, feeling devastated, low spirits, depression, and feelings of inadequacy: “*I was very upset and cried when I first heard about it. I felt very helpless*” (P02, female). Most participants also described experiencing a fearful acceptance process, including difficulty accepting the illness, disbelief, fear of it being fatal, and kinesiophobia. This fear was often rooted in unfamiliarity with the disease: “*I felt terrible. My morale was low. I thought it would be fatal because no one in my family had ever had it before*” (P10, male). Fear could also lead to avoidance of movement: “*I wasn’t moving because of the fear of attacks and fatigue*” (P04, female). Learning more about MS could help participants move towards acceptance: “*It was difficult to accept my illness at first, but then I calmed down a bit as I started to learn more*” (P07, female).

Daily life and role change: All participants reported difficulties with daily living activities such as cleaning, cooking, driving, and shopping, often contrasting what they used to do with what they could do now: “*Right now, I want to go and clean my house, but I can’t. I can’t clean the windows. I used to do my grocery shopping, but now I can’t*” (P05, female). One participant described adapting household tasks to her limited energy: “*For example, if I’m cooking and there’s something I need to do, like stirring the food in a pot, I bring a chair with me and do my work sitting on it. Generally, I can’t do it and I get tired*” (P08, female). Another factor causing difficulties in daily life was the inability to independently perform personal hygiene tasks such as going to the toilet, brushing teeth, and bathing, which was reported by some participants. Most participants stated that they experienced some role changes after the illness, particularly within the family and at work, and some had to use some assistive devices, such as a wheelchair or a urinary catheter, to maintain their independence: “*Things have changed a lot; I use a wheelchair. I need someone to support me*” (P12, female); “*I’ve started using a catheter*” (P11, female). These accounts show that the loss of independence was experienced not only as a physical limitation but also as a growing need for help from others.

Social Participation: Most participants reported that they had withdrawn from social life, whereas some described changes in their lifestyle due to the impact of multiple factors on their daily life activities. Withdrawal was often linked to reduced mobility: “*There have been many changes since this illness. My movements aren’t like they used to be. I’ve withdrawn more into the house*” (P03, female). Tasks previously performed by participants could also be taken over by family members: “*After shopping, I used to do everything myself. Now I can’t even carry the bags to the kitchen; my daughter carries them, unpacks them in the kitchen, and I put them away*” (P06, female). Others described reorganizing rather than abandoning their social lives: “*I live more planned now, I choose my social activities more carefully. I can’t say the quality has decreased, but I can say I’ve changed my life*” (P12, female).

Social Support: Most participants in our study reported receiving positive social support from their relatives, family, and friends, which they considered very valuable. Support was valued not only for practical and financial help but also because it made participants feel less alone and “normal”: “*My husband and children have been there for me through every difficulty, both financially and emotionally, and that’s what’s most effective for me*” (P02, female); “*Thanks to this support, I don’t feel alone. Most importantly, they make me feel ‘normal’*” (P05, female). Friendships with other individuals with MS were described as a distinctive source of understanding: “*Now I have friends with MS. I think we have a closer friendship because we understand each other. […] Just having them there is the biggest support for me*” (P05, female). In contrast, some individuals with MS stated that they were negatively affected by the negative attitudes of those around them, particularly when others did not understand invisible symptoms such as fatigue or when they increased participants’ fears: “*Those around me have difficulty understanding my symptoms, such as fatigue*” (P11, female); “*I think they scare me even more*” (P03, female).

#### 3.3.2. Expectations

Most participants looked to the future with hope, which was usually expressed as a wish for independence, strength, and less fatigue rather than for a cure: “*I want to get stronger, perhaps be able to move around without any physical support. Most importantly, I want to be independent*” (P07, female). One participant linked this hope directly to physiotherapy: “*I hope that in the future, thanks to physiotherapy, I will be able to live a completely independent life*” (P08, female). Notably, she also reported feeling worse after sessions (Section 3.4.1), which illustrates that hope and ambivalent treatment experiences could coexist. For others, hope was modest and focused on preserving their current condition: “*I hope it doesn’t get worse, I’m grateful for my current condition*” (P10, male). In contrast, some participants harbored anxieties about the future, anticipating further deterioration: “*I think I’ll get worse at this rate*” (P05, female). Anxiety about the future was associated with decreased independence, disease progression, and negative psychological states, whereas hope was associated with acceptance of the disease, appropriate treatment, preserved independence, the absence of role changes, and a better quality of life.

#### 3.3.3. Recommendations for Patients

Early physiotherapy: Most participants stated that they would advise newly diagnosed individuals with MS to begin physiotherapy programs as soon as they learn about their illness. These recommendations were grounded in their own experience that the benefits of physiotherapy are gradual and may consist of preventing deterioration rather than achieving visible improvement: “*Physiotherapy is a process; even if you don’t see results immediately, you will see the difference over time. Working with a good physiotherapist is very important*” (P05, female); “*When people say, ‘What good is physiotherapy if you haven’t gotten better?’ I say, ‘If I hadn’t gone to physiotherapy, I would have been worse off’*” (P03, female).

Coping strategies: Participants reported that seeking refuge in the Creator and the rapid acceptance and adaptation process positively affected the treatment and disease process; each was mentioned by some participants. Faith was expressed as a source of hope: “*I hope the creator will heal it, I believe*” (P05, female). For this participant, faith coexisted with active engagement in treatment, as she also strongly recommended physiotherapy to others. Participants also advised newly diagnosed individuals not to give up or withdraw: “*I would advise them not to dwell on the illness and not to restrict themselves from their social environment*” (P03, female); “*Keep going without forcing yourself, but also without giving up*” (P05, female).

Be active: Half of the participants stated that leading an active life is very valuable in living with the disease, while emphasizing that activity should be adjusted to one’s capacity: “*Walk and exercise from the beginning of the illness*” (P05, female); “*They should continue with a suitable sport or exercise program that doesn’t overexert them*” (P06, female).

### 3.4. Rehabilitation in Multiple Sclerosis

#### 3.4.1. Perception of Physiotherapy

Initial perception of physiotherapy: When participants were asked how they had perceived physiotherapy before their own treatment, two different views emerged: most participants stated that they had some knowledge of physiotherapy, whereas some had no knowledge of it: “*I had never heard of physiotherapy before*” (P01, female). Prior knowledge came from their own experience or from that of relatives, friends, or neighbors with other conditions, such as back and neck pain or stroke: “*My friends and family were going for back and neck pain*” (P11, female); “*Our neighbor had a stroke. A physiotherapist came to their house for treatment*” (P12, female). These accounts suggest that, before their own treatment, some participants associated physiotherapy with musculoskeletal problems or other neurological conditions rather than with MS.

Experience-based physiotherapy: Most participants reported that physiotherapy provided psychological, physical, and functional gains. Physical gains were described in terms of safer movement, fewer spasms, and more comfortable use of the limbs: “*Thanks especially to balance exercises and stretching movements, I feel safer and the spasms have decreased*” (P06, female); “*I think physiotherapy has been good for me, allowing me to use my arms and legs more comfortably and functionally*” (P07, female). Psychological and social gains were also emphasized: “*It is improving me psychologically*” (P11, female); “*It makes me feel better and more confident in social life*” (P01, female). However, not all experiences were positive. A few participants reported that physiotherapy did not provide any change: “*There has been no improvement. Everything continues the same*” (P04, female). One participant reported feeling worse after treatment because of increased fatigue: “*I think my energy has decreased even more after the treatments*” (P08, female).

Perceptual transformation: Participants in our study stated that their opinions and perceptions about physiotherapy changed over time. Most participants came to realize that physiotherapy was effective when continued regularly, and most described a change in their energy. Initial doubts were often replaced by a sense of benefit once participants had experienced the effects themselves: “*At first, I wasn’t sure if it would work. I was thinking, ‘I feel good when I’m inactive, why should I exercise?’ But after starting, I saw how right a decision it was. When I exercise, the fatigue decreases, and it becomes easier to move*” (P05, female). For some, the perceived benefit consisted of preventing deterioration rather than recovery: “*Before starting physiotherapy, I had concerns about whether it would work. Now I think it’s preventing my illness from getting worse*” (P10, male); “*People keep asking me if I’m benefiting from physical therapy. Think about it, a rolling stone gathers no moss; I’m constantly working out. Physical therapy is good for me; I get worse when I stay home*” (P09, male). Changes in energy were not uniformly positive; one participant described a short-lived benefit followed by fatigue: “*I feel fine for 40 min, but I feel bad after physiotherapy*” (P08, female). Participants whose perceptions did not change were those who could not attend physiotherapy regularly or who initially lacked sufficient information.

#### 3.4.2. Access to Rehabilitation

Facilitators: All participants described the physiotherapist’s positive attitude and the improvement in their physical and psychological well-being as factors that facilitated their participation in physiotherapy and rehabilitation programs; half mentioned ease of transportation and socialization, and some mentioned high motivation: “*Motivation is the most important thing*” (P12, female). The therapeutic relationship was central to continuing treatment, and for some participants the relationship itself was experienced as therapeutic: “*The most important factor in my continuing treatment is my physiotherapist’s friendly and compassionate nature*” (P03, female); “*I’m happier talking to my physiotherapist than the effects of the exercises. It’s good for me psychologically*” (P07, female). Participants also valued physiotherapists who explained the process and worked towards shared goals related to independence: “*My physiotherapist gave me detailed information about the process. We are working towards goals such as maintaining muscle strength, coping with fatigue, and improving balance. This is necessary for me to maintain my independence*” (P10, male). Practical arrangements and opportunities to socialize also supported attendance: “*Transportation is also easy because they pick me up from home with a shuttle service*” (P08, female); “*Before, I was always at home, but when I go to physiotherapy, it lifts my spirits, I make friends […]*” (P07, female).

Obstacles: All participants described their physical condition, such as fatigue, as a factor that could hinder participation; most mentioned inadequate transportation and a lack of information, some mentioned their psychological state, and a few mentioned a negative attitude of the physiotherapist. Physical and psychological barriers were closely intertwined, and their influence fluctuated from day to day: “*There are days when I don’t want to go to physical therapy because of fear of attacks and fatigue*” (P11, female); “*If I’m feeling down, I don’t want to go for treatment at all*” (P11, female). A lack of understanding in the social environment could undermine motivation: “*The lack of understanding from those around me upsets me a lot. This takes away my desire to continue physical therapy*” (P10, male). The physiotherapist’s attitude could act as a barrier as well as a facilitator: “*My physiotherapist’s grumpy face affects me very negatively*” (P11, female). Transportation difficulties could reduce the frequency of attendance: “*In the beginning, the physical therapy center I went to was very far from my home, and the shuttle service took a long detour, which made me nauseous, so I reduced it from 2 days a week to 1 day*” (P07, female). Paradoxically, feeling better could also lead to interruptions: “*Sometimes I want to stop physical therapy because I think I’m already fine. But then the old complaints return*” (P04, female). In addition, participants stated that they had not received sufficient guidance on rehabilitation from health professionals in the early stages of the disease.

Coping Strategies: Some individuals with MS developed their own strategies to cope with the symptoms of the disease. These included encouraging physical activity and practicing Pilates, yoga, and breathing exercises: “*Because I have difficulty using my arm and fingers, I try to do light weight exercises, ball squeezing and releasing, and regular walking on my own*” (P11, female); “*I watch yoga videos online to relax and try to practice them in my free time*” (P11, female). In contrast, half of the participants did not use any additional strategy outside their physiotherapy sessions: “*I don’t do anything. There are days when I don’t go to physical therapy, and I dedicate that day to rest*” (P01, female). These participants tended to be those who reported less social support.

Recommendations for rehabilitation: Participants made some suggestions to make their physiotherapy and rehabilitation treatments more effective. Most participants expected individual attention and individualized exercises, a functional approach including stretching, balance, and gait exercises, and a knowledgeable physiotherapist; most also recommended opening well-equipped centers and appointing competent physiotherapists. Some participants suggested that rehabilitation should also be possible remotely (telerehabilitation) and emphasized the need for support, and one participant had no suggestions. The demand for individualization was grounded in participants’ specific symptoms: “*Because of my vertigo, I shouldn’t do prone exercises. A physiotherapist should create a personalized exercise program for me*” (P10, male); “*Some rehabilitation centers don’t have physiotherapy specifically for MS. I would like more specialization and personalized programs in this area*” (P06, female). One group of participants argued against the use of weights: “*I can’t walk properly anyway, and it’s even harder when I have to use weights*” (P10, male). Some participants with MS indicated that bedside exercises were beneficial and should definitely be added to their program: “*Bedside exercises, especially those I do in the mornings, help me start the day more easily*” (P05, female). Stretching exercises, in particular, were described as very beneficial: “*Stretching exercises are especially good for me and my friends I’ve talked to. Why aren’t more stretching exercises done?*” (P12, female). Participants also considered the physiotherapist’s comprehensive knowledge and ability to share that knowledge with them to be important: “*My physiotherapist should be good in the field of MS and apply the correct treatment to me*” (P10, male). Some participants felt that physiotherapy sessions should not focus solely on physical well-being, as psychological improvement was also crucial.

## 4. Discussion

This qualitative study explored how individuals with RRMS experience living with disease and physiotherapy-based rehabilitation. Beyond confirming previously reported physical, psychological, and social effects of MS and known facilitators of and barriers to rehabilitation, our findings offer three insights. First, participants’ perceptions of physiotherapy were reshaped through their own treatment experience: initial unfamiliarity, the association of physiotherapy with other conditions, and doubts about its usefulness gave way, for most participants, to an understanding that its benefits are gradual, depend on continuity, and may consist of preventing deterioration rather than recovery. Second, the therapeutic relationship was experienced not only as a facilitator of attendance but also as a source of psychological benefit in its own right, whereas a physiotherapist’s negative attitude could discourage participation. Third, participation depended on an interplay of fluctuating symptoms, fear of attacks, mood, the understanding of the social environment, and practical arrangements such as transportation, so that the same participant could describe both facilitators and barriers. These insights were obtained in Türkiye, where most participants received long-term physiotherapy in private special education and rehabilitation centers, and they reflect participants’ perceptions rather than objective evidence of the effectiveness of physiotherapy.

The contribution of this study lies not in identifying entirely new barriers or facilitators of rehabilitation, but in illustrating how these factors intersect within individuals’ lived experiences. Participants’ accounts suggest that the perceived value of physiotherapy extends beyond immediate functional improvement to include the preservation of independence, psychological reassurance, and the development of more realistic expectations regarding rehabilitation. These findings highlight the importance of understanding rehabilitation engagement as a dynamic and context-dependent experience rather than solely as adherence to a prescribed exercise program.

### 4.1. Living with Multiple Sclerosis

#### 4.1.1. Multidimensional Effects

The physical, psychological, and social effects reported by individuals with MS in our study are consistent with the literature. Sensory-motor problems, speech disorders, fatigue, balance and walking difficulties, pain, and bladder dysfunction are frequently reported, as well as stress, anxiety, loneliness, concealment or denial of the disease, and loss of social function [4,6]. The shock, helplessness, and hopelessness experienced during the diagnostic process also support the findings of emotional distress in our study [33]; similarly, a meta-synthesis of qualitative studies described receiving the diagnosis as an emotional upheaval involving shock, denial, anger, and fear, and identified acceptance as an adaptive response [17]. In our study, fear of a fatal outcome and fear of movement appeared to be fueled by unfamiliarity with the disease, and learning more about MS could ease this distress, which underlines the importance of providing disease-specific information at the time of diagnosis.

MS can lead to changes in daily living roles and the need for physical or psychological support [5,34]. Problems such as fatigue, physical weakness, balance problems, and muscle stiffness have been reported to limit daily living activities [5], and the disease has been reported to affect identity perception, work, and family life [7,20,23]. Similar role changes were observed in our study; our findings further suggest that these changes were experienced as a loss of independence and a growing reliance on others, which may explain why independence was central to participants’ hopes for the future and to the goals they valued in physiotherapy.

Participants emphasized the importance of social support regardless of positive or negative experiences, and a systematic review identified social support as a protective factor for quality of life in adults with MS [35]. Individuals who do not receive support from their immediate environment and experience feelings of pity, exclusion, or being ignored are reported to experience increased disappointment, anger, and loneliness [17,21,23]. Conversely, positive social support is thought to support adaptation to illness, coping, and emotional well-being [21,36]. Our findings add that support was valued particularly when it made participants feel understood and “normal”, that peers with MS offered a distinctive form of mutual understanding, and that a lack of understanding of invisible symptoms such as fatigue not only caused distress but could also reduce motivation to continue physiotherapy.

Some participants in our study reported difficulties in performing personal hygiene tasks independently and the need for assistive devices, including a wheelchair and a urinary catheter. The experiences of individuals with MS who use clean intermittent catheterization have only recently been examined qualitatively [37], and our findings suggest that such needs should also be considered in rehabilitation programs that target independence in daily living.

#### 4.1.2. Recommendations for Patients

Participants in our study described different coping strategies and advised newly diagnosed individuals not to give up, not to lose hope, and not to withdraw from their social environment; similarly, individuals newly diagnosed with MS have been reported to try to keep their lives as normal as possible and to regain hope and a sense of control [33]. Acceptance has been reported to be the coping strategy most frequently used by individuals with MS [38], and in our study, rapid acceptance and adaptation were likewise perceived as positively affecting the treatment and disease process. Being positive and having a positive mindset, that is, not losing hope, were among the results that were consistent with the literature [15]. Using religious belief to cope with MS, and a feeling of complete surrender to the Creator, were also among the suggestions made by participants. In a study of individuals with MS in Poland, a religious meaning system was associated with higher life satisfaction through a greater sense of meaning in life [39]. In our study, faith was expressed as hope alongside, rather than instead of, active engagement in physiotherapy. Thus, faith-based coping may be related to a more accepting approach and greater hope, and its role in rehabilitation deserves further exploration in culturally diverse samples.

In a qualitative study of individuals in the early stages of MS (mean time since diagnosis, approximately 2 years), most participants reported that their level of disability did not require rehabilitation [22]. This contradicts the participants in our study who believed that physiotherapy should begin as early as possible. This difference may be explained by disease stage and experience: our participants had a longer disease duration, moderate to severe disability, and long-term physiotherapy experience, and their recommendation for early physiotherapy was grounded in having experienced its gradual benefits and the deterioration they associated with inactivity.

#### 4.1.3. Expectations

In our study, most individuals with MS were hopeful about the future, while some approached it with anxiety, which was associated with decreased independence and disease progression (Section 3.3.2). This finding is consistent with the literature [5,7,19,40]. In particular, hope in our study was oriented towards independence and the preservation of current function rather than towards a cure, which is consistent with reports that positive future-oriented cognitions are associated with better quality of life in MS [40]; physiotherapy itself could be a source of this hope.

### 4.2. Rehabilitation in Multiple Sclerosis

#### 4.2.1. Perception of Physiotherapy

In our study, most participants were knowledgeable about physiotherapy and rehabilitation and were familiar with these services through their own experience or that of their relatives. This finding differs from a study that reported that a lack of awareness limited access to services [11]. This difference may be explained by the fact that our participants were recruited from a hospital neurology clinic, were all receiving physiotherapy, and had more contact with healthcare services. A previous qualitative study reported that physiotherapy was more associated with orthopedic problems and advanced neurological diseases [22]. Our participants’ initial perceptions, which were shaped by relatives’ and neighbors’ experiences of back pain or stroke, resemble this association; however, their later recommendations for early physiotherapy suggest that their own treatment experience led them to recognize its protective and preventive role.

Most participants stated that their perceptions of physiotherapy changed positively over time, often after initial doubts about whether it would work; similarly, some participants in an interview study on inpatient MS rehabilitation described fundamental changes in perspective [13]. In our study, initial doubts were associated with insufficient information and, for some participants, with not yet experiencing significant physical effects, whereas peer interaction, physical and psychological gains, and the reorganization of life may have increased the value given to the process.

Psychological benefits were also the most frequently mentioned achievements of rehabilitation in a previous interview study [13]. In our study, most participants reported that physiotherapy improved their psychological and physical well-being and independence in daily living; for some, the psychological benefit derived as much from the relationship with the physiotherapist and from socializing as from the exercises themselves. However, some participants perceived no change or felt worse after sessions. Importantly, the presence of neutral and unfavorable experiences suggests that physiotherapy should not be assumed to be uniformly beneficial from the perspective of individuals with MS. Reports of no perceived improvement or feeling worse after sessions highlight the need to consider individual symptom fluctuations, treatment expectations, and perceived tolerability when interpreting rehabilitation experiences. These accounts reinforce the importance of regularly reassessing individuals’ goals and experiences rather than assuming that continued attendance necessarily reflects perceived benefit.

#### 4.2.2. Access to Rehabilitation

A previous qualitative study reported that a physiotherapist experienced in MS increased participants’ trust in and engagement with rehabilitation [22]. This finding is consistent with our study’s identification of the therapist’s positive and empathetic attitude as facilitating and their negative attitude as hindering. For some participants, the interaction with the physiotherapist was itself psychologically beneficial, which may be particularly relevant when social participation has narrowed; therapeutic relationships, communication, and peer support have likewise been reported to support rehabilitation [23,41]. However, usual-care physiotherapy did not change self-efficacy or physical activity in a longitudinal study, which recommended integrating motivational strategies [42].

The main obstacles to rehabilitation in our study, namely transportation difficulties, pain, fatigue, low physical capacity, anxieties about disease worsening, and psychological problems, are consistent with previous studies [23,24,43], and a lack of knowledge specific to MS and limited access to specialized care have been reported to negatively affect quality of life and the benefit from rehabilitation [23]. In line with our participants’ report of insufficient early guidance from healthcare professionals, only four of nine participants in a previous study who had received MS-specific rehabilitation reported that their neurologist had recommended it [22]. The organization of services, such as transport arrangements, and education about the value of continuity may therefore be as important for participation as the exercise program itself.

Regarding sociodemographic and cultural background, physiotherapy use in a large Swiss registry-based study was associated mainly with disability level, progressive MS, and gait problems, with little evidence of sociodemographic barriers [44], whereas a qualitative study called for research on racial, ethnic, and cultural influences on rehabilitation experiences [22]. Most of our participants attended private centers that provide state-funded physiotherapy and usually offer transportation, which may have reduced financial barriers, although transportation difficulties were still frequently reported. Our small, relatively homogeneous sample did not allow us to examine the influence of education, employment, or marital status on access, which should be addressed in larger and more diverse samples.

Participants emphasized the importance of balance and strengthening exercises in addition to regular rehabilitation; exercise has been reported to improve functional outcomes, including balance and walking [14,45]. Stretching was also considered beneficial, although evidence that exercise improves flexibility in MS is still preliminary [46], and the perceived usefulness of bedside exercises is consistent with recommendations to adapt exercise to the level of disability [47]. Some participants also practiced yoga, Pilates, and breathing exercises, whose positive effects have been reported previously [48,49,50]; such physical activity and mind–body-based practices can be used as active coping strategies [4].

The participants’ suggestion of telerehabilitation to reduce transportation barriers also aligns with previous studies [8], although the evidence for telerehabilitation has been rated as low quality [14]. Their requests for programs individualized to symptoms such as vertigo and walking difficulties are consistent with recommendations for personalized, patient-centered, and goal-oriented rehabilitation [8,10,12], which may support not only motor functions but also psychological and social well-being.

Overall, our findings are consistent with qualitative evidence describing life with MS as a dynamic adaptation process involving information seeking, uncertainty, changing life roles and identities, and the impact of fatigue on daily life and relationships [18]. Our study suggests that the MS experience is shaped not only by symptoms but also by rehabilitation perception, expectations, and environmental conditions, and that, from the participants’ perspective, physiotherapy-based rehabilitation can become part of this adaptation process when it is accessible, individualized, and delivered within a supportive therapeutic relationship.

### 4.3. Strengths and Limitations

The main strength of this study is that it examined the illness and physiotherapy experiences of individuals with MS together and supported the findings with verbatim quotations. However, the participants were a small, self-selected group recruited from a single hospital, and all had RRMS; therefore, the findings should be transferred to other clinical and institutional contexts with caution. An additional limitation is that all participants were receiving physiotherapy at the time of the interviews. Consequently, the sample may overrepresent individuals who were willing and able to engage with rehabilitation services and may underrepresent those who discontinued treatment, declined rehabilitation, or experienced substantial barriers to accessing care. This selection characteristic should be considered when interpreting the predominantly favorable perceptions of physiotherapy. Furthermore, although code saturation was observed after the tenth interview, this should not be interpreted as evidence that the full complexity of participants’ experiences was captured. Code saturation indicates that no additional codes were identified in the final interviews, whereas meaning saturation requires a more comprehensive understanding of the nuances and variations within identified themes. Given the relatively small and clinically specific sample, some dimensions of participants’ experiences may remain unexplored. In addition, the findings reflect participants’ perceptions and should not be interpreted as objective evidence of the effectiveness of physiotherapy. Some interview questions may have presupposed positive effects, and the influence of the interviewer cannot be excluded, although negative and neutral experiences were also reported. Finally, referral pathways and telerehabilitation were not explored in depth.

## 5. Conclusions

This study suggests that individuals with MS experience physiotherapy-based rehabilitation not merely as an intervention, but as a process of coping with the disease, regaining functions, making sense of uncertainty, and increasing social participation. Rather than identifying new phenomena, the study deepens existing knowledge by showing that participants’ perceptions of physiotherapy changed through their own experience towards an appreciation of gradual benefits that depend on continuity and may consist of preventing deterioration; that the therapeutic relationship was a source of psychological benefit or, when negative, a barrier; and that participation depended on fluctuating symptoms, psychological state, the understanding of others, and transportation. These perception-based findings do not establish the effectiveness of physiotherapy.

For physiotherapists, this means that individuals with MS should be provided with clear, reliable, and disease-specific rehabilitation information from an early stage, including the aims and possibly gradual, preventive benefits of physiotherapy, and that programs should be individualized to symptoms such as fatigue, vertigo, and walking difficulties, address psychological well-being within an empathetic therapeutic relationship, and help individuals manage barriers to continuity. In addition, a stronger communication network should be established among neurologists, physiotherapists, and other healthcare professionals to support earlier referral. Future studies should include individuals with progressive MS and those not receiving physiotherapy, examine referral pathways and telerehabilitation, and combine qualitative findings with clinical outcome measures.

## Figures and Tables

**Table 1:** Semi-Structured Interview Form for Physiotherapy Experiences of Individuals with Multiple Sclerosis.

General Health Status and MS Experience	How did you feel when you received your MS diagnosis? What changes occurred in your life during this process?
	How do you think MS affects your daily life? What activities do you find difficult? (Daily living activities, quality of life, etc.)
	How do you feel physically, emotionally, and socially during the illness? (Fatigue, sleep, pain, etc.)
Perspectives on Physiotherapy	Do you think physiotherapy is effective in reducing the symptoms you experience with MS?
	How much do you know about your physiotherapy treatment? What are your expectations from physiotherapy?
	What were your thoughts about physiotherapy before starting the treatment? Did your thoughts change after you started?
Effects and Expectations of Physiotherapy	Which physiotherapy techniques (e.g., bed exercises, balance exercises, gait exercises, relaxation techniques) do you find most beneficial for you?
	What can you say about the effect of physiotherapy on disease symptoms or your overall health?
	In what areas did you notice you felt better when you regularly continued with physiotherapy?
	What factors influence participation in physiotherapy sessions (motivation, transportation, energy level, etc.)?
	What factors positively influence the physiotherapy process?
	What factors negatively affect the physiotherapy process?
	What are your expectations from your physiotherapist during the physiotherapy process?
	What do you think are the challenges encountered during the rehabilitation process?
Quality of Life and Social Support	How do you think your quality of life has changed since having MS?
	Do you think physiotherapy affects your participation in social and professional life? To what extent?
	How does the support you receive from family and friends help you cope with the illness?
Physical Activity and Mobility	How has your physical activity level changed since you were diagnosed with MS?
	Did your participation in physical activity increase after starting physiotherapy? If so, in which areas?
	How has physiotherapy affected your daily mobility or the way you perform physical activities?
	What are the biggest challenges you face during physical activities? Does physiotherapy help you overcome these challenges?
	Besides physiotherapy, do you use any other strategies to increase physical activity?
	Do you think physiotherapy increases or decreases your energy and endurance? In which activities did you notice a difference?
Future Expectations and Recommendations	Based on the benefits and results you’ve received from physiotherapy, what are your expectations for the future?
	Is there anything you would like to see changed in your MS treatment or physiotherapy processes?
	What are your recommendations for individuals diagnosed with MS and their physiotherapy process?

**Table 2:** Clinical and rehabilitation characteristics of participants.

Participant	Gender	Age (Years)	EDSS	Disease Duration (Years)	Physiotherapy Setting	Physiotherapy Duration (Years)
**P01**	Female	38	4	3	Private center	1
**P02**	Female	42	6	2.5	Private center	1
**P03**	Female	45	3	10	Private center	5
**P04**	Female	48	3	2	Hospital	1
**P05**	Female	53	4	3	Private center	1
**P06**	Female	55	5	7.5	Private center	4
**P07**	Female	44	6	9	Private center	2
**P08**	Female	36	7	7	Private center	3
**P09**	Male	39	6	8	Private center	5
**P10**	Male	47	7	10.5	Private center	7
**P11**	Female	45	3	3	Hospital	1
**P12**	Female	55	4	8	Private center	6

EDSS: Expanded Disability Status Scale.

## Data Availability

The datasets used and/or analysed during the current study are available from the corresponding author on reasonable request, subject to ethical and confidentiality considerations. The datasets are not publicly available because they contain sensitive qualitative information regarding participants’ personal experiences and health conditions, and public disclosure could potentially compromise participant confidentiality.

## Supplementary Materials

The following supporting information can be downloaded at: https://www.mdpi.com/article/10.3390/healthcare14213788/s1, Supplementary S1: COREQ (Consolidated Criteria for Reporting Qualitative Research) checklist; Supplementary S2: Summary of participants’ responses to the semi-structured interview questions (representative quotations). Reference [31] is cited in the Supplementary Materials.

## Abbreviations

The following abbreviations are used in this manuscript:

MS	Multiple sclerosis
RRMS	Relapsing–remitting multiple sclerosis
EDSS	Expanded Disability Status Scale
COREQ	Consolidated Criteria for Reporting Qualitative Research
