# Supplementary material for: Physiotherapy-Based Rehabilitation from the Perspective of Individuals with Multiple Sclerosis: Independence and Psychological Well-Being Beyond Physical Function—A Qualitative Study
Source: Healthcare (Basel). 2026 Nov 6;14(21):3788. doi: 10.3390/healthcare14213788 (PMC13762594; doi:10.3390/healthcare14213788)
Supplement: Supplementary file 1 [file healthcare-14-03788-s001.zip › Supplementary S1 COREQ Checklist.pdf]

Supplementary S1. Consolidated Criteria for Reporting Qualitative Research (COREQ) checklist

Physiotherapy-Based Rehabilitation from the Perspective of Individuals with Multiple Sclerosis: Independence and Psychological Well-being Beyond Physical Function—A Qualitative Study

Checklist items and guide questions are from Tong, A.; Sainsbury, P.; Craig, J. Consolidated criteria for reporting qualitative research (COREQ): A 32-item checklist for interviews and focus groups. Int. J. Qual. Health Care 2007, 19, 349–357. https://doi.org/10.1093/intqhc/mzm042. Line numbers refer to the revised manuscript.

No.	Item	Guide question/description	Reported in this study	Location in the revised manuscript
**Domain 1: Research team and reflexivity**				
Personal characteristics				
1	**Interviewer/facilitator**	Which author/s conducted the interview or focus group?	All interviews were conducted by M.B.	Section 2.3 (line 184)
2	**Credentials**	What were the researcher's credentials? E.g., PhD, MD	PhD; academic title: assistant professor	Section 2.3 (lines 184–185)
3	**Occupation**	What was their occupation at the time of the study?	Assistant professor, Department of Physiotherapy and Rehabilitation	Section 2.3 (lines 184–185)
4	**Gender**	Was the researcher male or female?	Male	Section 2.3 (line 184)
5	**Experience and training**	What experience or training did the researcher have?	Clinical and academic experience in neurological rehabilitation	Section 2.3 (lines 185–186)
Relationship with participants				
6	**Relationship established**	Was a relationship established prior to study commencement?	No; the interviewer had no prior relationship with the participants	Section 2.3 (lines 186–187)
7	**Participant knowledge of the interviewer**	What did the participants know about the researcher? E.g., personal goals, reasons for doing the research	Participants received a brief explanation of the purpose of the study; the interview questions were not shared in advance	Section 2.4 (lines 202–203)
8	**Interviewer characteristics**	What characteristics were reported about the interviewer/facilitator? E.g., bias, assumptions, reasons and interests in the research topic	Possible assumptions arising from the researchers' professional backgrounds (expectation of positive effects of physiotherapy; possible tendency of participants to report favorable experiences) and the strategies used to limit their influence	Section 2.3 (lines 187–194)

No.	Item	Guide question/description	Reported in this study	Location in the revised manuscript
**Domain 2: Study design**				
Theoretical framework				
9	**Methodological orientation and theory**	What methodological orientation was stated to underpin the study? E.g., grounded theory, discourse analysis, ethnography, phenomenology, content analysis	Descriptive phenomenological orientation; inductive thematic analysis undertaken within a descriptive phenomenological framework (six phases of Braun and Clarke)	Section 2.1 (lines 130–145)
Participant selection				
10	**Sampling**	How were participants selected? E.g., purposive, convenience, consecutive, snowball	Purposive sampling	Section 2.2 (line 152)
11	**Method of approach**	How were participants approached? E.g., face-to-face, telephone, mail, email	Eligible individuals were invited to participate by a neurologist working in the neurology outpatient clinic of Fırat University Hospital	Section 2.2 (lines 152–155)
12	**Sample size**	How many participants were in the study?	12 participants	Section 2.2 (lines 175–177)
13	**Non-participation**	How many people refused to participate or dropped out? Reasons?	Of the 18 individuals contacted, 3 declined to participate; of the 15 who agreed, 3 withdrew before the interview because of time constraints and the long distance to the interview location	Section 2.2 (lines 173–177)
Setting				
14	**Setting of data collection**	Where was the data collected? E.g., home, clinic, workplace	Face-to-face interviews in a room in which only the participant and the interviewer were present	Section 2.4 (lines 206–207)
15	**Presence of non-participants**	Was anyone else present besides the participants and researchers?	No	Section 2.4 (lines 206–207)
16	**Description of sample**	What are the important characteristics of the sample? E.g., demographic data, date	Gender, age, MS type, EDSS, disease duration, marital status, education, employment, and physiotherapy setting and duration; interviews were conducted between 1 January and 1 December 2025	Section 3.1 (lines 236–251), Table 2; Section 2.4
Data collection				
17	**Interview guide**	Were questions, prompts, guides provided by the authors? Was it pilot tested?	Yes; the semi-structured interview guide is provided in Table 1. Pilot testing of the guide is not reported	Section 2.4 (lines 196–201); Table 1

No.	Item	Guide question/description	Reported in this study	Location in the revised manuscript
18	**Repeat interviews**	Were repeat interviews carried out? If yes, how many?	No	Section 2.2 (line 181)
19	**Audio/visual recording**	Did the research use audio or visual recording to collect the data?	Audio recording (smartphones), with the written consent of the participants	Section 2.4 (lines 205–206)
20	**Field notes**	Were field notes made during and/or after the interview or focus group?	Not reported; the analysis was based on the verbatim transcripts	—
21	**Duration**	What was the duration of the interviews or focus group?	30–40 minutes	Section 2.4 (line 207)
22	**Data saturation**	Was data saturation discussed?	Yes; saturation was assessed at the code level: code saturation was reached after the 10th interview, and no new codes emerged in the 11th and 12th interviews. The distinction between code saturation and meaning saturation is acknowledged as a limitation	Section 2.2 (lines 177–181); Section 4.3 (lines 691–698)
23	**Transcripts returned**	Were transcripts returned to participants for comment and/or correction?	No	Section 2.2 (lines 181–182)
**Domain 3: Analysis and findings**				
Data analysis				
24	**Number of data coders**	How many data coders coded the data?	Two researchers (S.E.B. and M.B.), independently	Section 2.5 (lines 217–218)
25	**Description of the coding tree**	Did authors provide a description of the coding tree?	Yes; overarching themes, themes, subthemes, and codes are shown in Figure 1 and Table 3	Section 3.2; Table 3; Figure 1
26	**Derivation of themes**	Were themes identified in advance or derived from the data?	Derived from the data (inductive analysis; no predefined coding framework)	Section 2.5 (lines 222–224)
27	**Software**	What software, if applicable, was used to manage the data?	MAXQDA 2020.2.2 (VERBI Software, Berlin, Germany)	Section 2.5 (line 222)
28	**Participant checking**	Did participants provide feedback on the findings?	No; transcripts were not returned to participants, and no participant feedback on the findings is reported	Section 2.2 (lines 181–182)

No.	Item	Guide question/description	Reported in this study	Location in the revised manuscript
Reporting				
29	**Quotations presented**	Were participant quotations presented to illustrate the themes/findings? Was each quotation identified? E.g., participant number	Yes; each quotation is identified by participant number and gender	Sections 3.3–3.4; Table 3; Supplementary 1
30	**Data and findings consistent**	Was there consistency between the data presented and the findings?	Yes; each subtheme is presented with the corresponding codes, the number of participants, and quotations	Sections 3.2–3.4; Table 3
31	**Clarity of major themes**	Were major themes clearly presented in the findings?	Yes; two overarching themes and five themes	Section 3.2; Figure 1
32	**Clarity of minor themes**	Is there a description of diverse cases or discussion of minor themes?	Yes; 17 subthemes are described, including negative and divergent cases (e.g., no perceived change, feeling worse after sessions, a negative attitude of the physiotherapist)	Sections 3.3–3.4
