# Supplementary material for: Physiotherapy-Based Rehabilitation from the Perspective of Individuals with Multiple Sclerosis: Independence and Psychological Well-Being Beyond Physical Function—A Qualitative Study
Source: Healthcare (Basel). 2026 Nov 6;14(21):3788. doi: 10.3390/healthcare14213788 (PMC13762594; doi:10.3390/healthcare14213788)
Supplement: Supplementary file 1 [file healthcare-14-03788-s001.zip › Supplementary S2.pdf]

Supplementary S2. Summary of Participants' Responses to the Semi-Structured Interview Questions

Category	Theme	Subtheme	Patients	Representative quote
Living with Multipl Sclerosis	Multidimensional effects	Physical impact	P01	I experience a lot of pain and spasms. I have sleep problems because of the pain.
			P09	I experience extreme fatigue and need more sleep
			P03	Depending on the frequency of my attacks, there have been days when I couldn't even get out of bed.
			P02	I have difficulty walking outside; I can only walk with assistance and support.
			P11	Initially, I wasn't physically affected much, but as the disease progressed, my hand became numb, I couldn't do work, and I had balance problems.
			P10	I have difficulties walking, talking, and adapting to social situations.
		Emotional impact	P07	It was difficult to accept my illness at first, but then I calmed down a bit as I started to learn more
			P02	I was very upset and cried when I first heard about it. I felt very helpless.
			P11	My mental state was devastated.
			P10	I felt terrible. My morale was low. I thought it would be fatal because no one in my family had ever had it before.
			P04	I wasn't moving because of the fear of attacks and fatigue.
		Daily life and role changes	P08	For example, if I'm cooking and there's something I need to do, like stirring the food in a pot, I bring a chair with me and do my work sitting on it. Generally, I can't do it and I get tired
			P03	I can't write because I have difficulty holding a pen
			P11	I used to be able to go out and shop, but I can't anymore
			P05	Right now, I want to go and clean my house, but I can't. I can't clean the windows. I used to do my grocery shopping, but now I can't
			P12	Things have changed a lot; I use a wheelchair. I need someone to support me.
			P11	I've started using a catheter.
		Social participation	P03	I can't travel, I can't go to the places I want to go. My children want to take me places, but I can't.
			P06	I live a more introverted life in my social life, but even though I have MS, my zest for life hasn't ended.
			P03	There have been many changes since this illness. My movements aren't like they used to be. I've withdrawn more into the house.
			P07	My quality of life has decreased a bit, but I haven't completely cut myself off from social life.
			P12	I live more planned now, I choose my social activities more carefully. I can't say the quality has decreased, but I can say I've changed my life.
			P06	After shopping, I used to do everything myself. Now I can't even carry the bags to the kitchen; my daughter carries them, unpacks them in the kitchen, and I put them away
		Social support	P05	Now I have friends with MS. I think we have a closer friendship because we understand each other. Sometimes I feel devastated because of the pain, and my friend is there for me and says it will pass. Just having them there is the biggest support for me.
			P02	My husband and children have been there for me through every difficulty, both financially and emotionally, and that's what's most effective for me
			P05	Thanks to this support, I don't feel alone. Most importantly, they make me feel 'norma
			P07	Talking and chatting with them helps me open up, distracts me, clears my mind, and relieves my worries. I like to share; I feel suffocated when I don't talk
			P04	Nowadays, everyone is preoccupied with their own problems
			P03	I think they scare me even more
			P09	My family and friends aren't supportive

			P11	Those around me have difficulty understanding my symptoms, such as fatigue.
	Expectations	Hope for the future	P08	I hope that in the future, thanks to physiotherapy, I will be able to live a completely independent life
			P12	I envision spending my days with less fatigue in the future
			P10	I hope it doesn't get worse, I'm grateful for my current condition
			P07	I want to get stronger, perhaps be able to move around without any physical support. Most importantly, I want to be independent
		Anxiety about the future	P03	I don't think I'll get any better than this
			P05	I think I'll get worse at this rate.
	Recommendations for patients	Early physiotherapy	P03	When people say, 'What good is physiotherapy if you haven't gotten better?' I say, 'If I hadn't gone to physiotherapy, I would have been worse off.'
			P02	They absolutely need to do physical therapy; it's essential for an active and mobile social life
			P05	Physiotherapy is a process; even if you don't see results immediately, you will see the difference over time. Working with a good physiotherapist is very important
			P07	They should go to physical therapy. I tell them that already. My sister-in-law is also ill, and I tell her to get treatment too; I'm happy with it, and I recommend that she come as well
		Coping strategies	P03	I would advise them not to dwell on the illness and not to restrict themselves from their social environment
			P10	Don't get discouraged
			P05	Keep going without forcing yourself, but also without giving up
			P07	They should never lose hope, they should continue with life, pay attention to healthy eating, and most importantly, they should focus on recovery and make an effort throughout the illness
			P05	I hope the creator will heal it, I believe.
		Be active	P05	Walk and exercise from the beginning of the illness
			P03	Do everything you can to lead an active life
			P06	they should continue with a suitable sport or exercise program that doesn't overexert them
Rehabilitation in Multiple Sclerosis	Perception of Physiotherapy	Initial perception of physiotherapy	P01	I had never heard of physiotherapy before
			P11	My friends and family were going for back and neck pain
			P12	Our neighbor had a stroke. A physiotherapist came to their house for treatment
		Experience based physiotherapy	P06	Thanks especially to balance exercises and stretching movements, I feel safer and the spasms have decreased
			P07	I think physiotherapy has been good for me, allowing me to use my arms and legs more comfortably and functionally.
			P11	It is improving me psychologically.
			P01	It makes me feel better and more confident in social life
			P03	I've become a little more comfortable with housework compared to the beginning
			P04	There has been no improvement. Everything continues the same.
			P08	I think my energy has decreased even more after the treatments
		Perceptual transformation	P07	Not at first, but later I felt my muscles becoming more flexible, and I noticed that my walking and balance were better
			P09	People keep asking me if I'm benefiting from physical therapy. Think about it, a rolling stone gathers no moss.; I'm constantly working out. Physical therapy is good for me; I get worse when I stay home
			P05	At first, I wasn't sure if it would work. I was thinking, 'I feel good when I'm inactive, why should I exercise?' But after starting, I saw how right a decision it was. When I exercise, the fatigue decreases, and it becomes easier to move.

			P07	Before, I was always at home, but when I go to physiotherapy, it lifts my spirits, I make friends, and the physiotherapists with me
			P08	I feel fine for 40 minutes, but I feel bad after physiotherapy
			P10	Before starting physiotherapy, I had concerns about whether it would work. Now I think it's preventing my illness from getting worse.
	Access to rehabilitation	Facilitators	P12	Motivation is the most important thing
			P03	The most important factor in my continuing treatment is my physiotherapist's friendly and compassionate nature
			P07	I'm happier talking to my physiotherapist than the effects of the exercises. It's good for me psychologically
			P08	Transportation is also easy because they pick me up from home with a shuttle service
			P10	My physiotherapist gave me detailed information about the process. We are working towards goals such as maintaining muscle strength, coping with fatigue, and improving balance. This is necessary for me to maintain my independence
			P11	Her friendly, positive energy and knowledge of the exercises positively affect my continuity
		Obstacles	P11	"If I'm feeling down, I don't want to go for treatment at all
			P10	The lack of understanding from those around me upsets me a lot. This takes away my desire to continue physical therapy.
			P04	Sometimes I want to stop physical therapy because I think I'm already fine. But then the old complaints return
			P11	"There are days when I don't want to go to physical therapy because of fear of attacks and fatigue
			P11	My physiotherapist's grumpy face affects me very negatively.
			P07	In the beginning, the physical therapy center I went to was very far from my home, and the shuttle service took a long detour, which made me nauseous, so I reduced it from 2 days a week to 1 day
		Coping strategies	P11	Because I have difficulty using my arm and fingers, I try to do light weight exercises, ball squeezing and releasing, and regular walking on my own
			P02	I set daily step goals for myself
			P08	I started doing light Pilates. I do breathing exercises
			P11	I watch yoga videos online to relax and try to practice them in my free time
			P02	I don't do anything. I'm not that active at home
			P01	I don't do anything. There are days when I don't go to physical therapy, and I dedicate that day to rest
		Recommendations for rehabilitation	P10	Because of my vertigo, I shouldn't do prone exercises. A physiotherapist should create a personalized exercise program for me.
			P06	Some rehabilitation centers don't have physiotherapy specifically for MS. I would like more specialization and personalized programs in this area.
			P01	Also, having personalized exercise programs makes the process easier
			P05	Bedside exercises, especially those I do in the mornings, help me start the day more easily
			P10	I can't walk properly anyway, and it's even harder when I have to use weights
			P11	I have a lot of difficulty walking and climbing stairs in daily life. For this reason, balance exercises, walking, and stair climbing should be worked on more

			P12	Stretching exercises are especially good for me and my friends I've talked to. Why aren't more stretching exercises done?
			P10	My physiotherapist should be good in the field of MS and apply the correct treatment to m
